# Health Benefits and Pharmacological Properties of Carvone

**DOI:** 10.3390/biom11121803

**Published:** 2021-12-01

**Authors:** Abdelhakim Bouyahya, Hamza Mechchate, Taoufiq Benali, Rokia Ghchime, Saoulajan Charfi, Abdelaali Balahbib, Pavel Burkov, Mohammad Ali Shariati, Jose M. Lorenzo, Nasreddine El Omari

**Affiliations:** 1 Laboratory of Human Pathologies Biology, Department of Biology, Faculty of Sciences, and Genomic Center of Human Pathologies, Faculty of Medicine and Pharmacy, Mohammed V University in Rabat, Rabat 10106, Morocco; 2 Laboratory of Biotechnology, Environment, Agri-Food, and Health (LBEAS), Faculty of Sciences, University Sidi Mohamed Ben Abdellah (USMBA), Fez B.P. 1796, Morocco; hamza.mechchate@usmba.ac.ma; 3 Environment and Health Team, Polydisciplinary Faculty of Safi, Cadi Ayyad University, Sidi Bouzid B.P. 4162, Morocco; benali.taoufiq@gmail.com; 4 Department of Clinical Neurophysiology, Hospital of Specialities, Ibn Sina University Hospital, Rabat B.P 6527, Morocco; Rabat rokairo@gmail.com; 5 Laboratory of Biotechnology and Applied Microbiology, Department of Biology, Faculty of Sciences, Abdelmalek Essaadi University, Tetouan B.P. 2117, Morocco; sawlajan@gmail.com; 6 Laboratory of Biodiversity, Ecology and Genome, Faculty of Sciences, Mohammed V University, Rabat 10106, Morocco; balahbib.abdo@gmail.com; 7 South Ural State Agrarian University, 13 Gagarina St., 457100 Troitsk, Russia; burcovpavel@mail.ru; 8 Research Department, K.G. Razumovsky Moscow State University of Technologies and Management (The First Cossack University), 73, Zemlyanoy Val St., 109004 Moscow, Russia; shariatymohammadali@gmail.com; 9 Centro Tecnológico de la Carne de Galicia, Rúa Galicia Nº 4, Parque Tecnológico de Galicia, San Cibrao das Viñas, 32900 Ourense, Spain; 10 Área de Tecnología de los Alimentos, Facultad de Ciencias de Ourense, Universidad de Vigo, 32004 Ourense, Spain; 11 Laboratory of Histology, Embryology, and Cytogenetic, Faculty of Medicine and Pharmacy, Mohammed V University in Rabat, Rabat 10100, Morocco; nasrelomari@gmail.com

**Keywords:** carvone, pharmacological properties, mechanism of action

## Abstract

Carvone is a monoterpene ketone contained in the essential oils of several aromatic and medicinal plants of the Lamiaceae and Asteraceae families. From aromatic plants, this monoterpene is secreted at different concentrations depending on the species, the parts used, and the extraction methods. Currently, pharmacological investigations showed that carvone exhibits multiple pharmacological properties such as antibacterial, antifungal, antiparasitic, antineuraminidase, antioxidant, anti-inflammatory, and anticancer activities. These studies were carried out in vitro and in vivo and involved a great deal of knowledge on the mechanisms of action. Indeed, the antimicrobial effects are related to the action of carvone on the cell membrane and to ultrastructural changes, while the anti-inflammatory, antidiabetic, and anticancer effects involve the action on cellular and molecular targets such as inducing of apoptosis, autophagy, and senescence. With its multiple mechanisms, carvone can be considered as natural compounds to develop therapeutic drugs. However, other investigations regarding its precise mechanisms of action as well as its acute and chronic toxicities are needed to validate its applications. Therefore, this review discusses the principal studies investigating the pharmacological properties of carvone, and the mechanism of action underlying some of these properties. Moreover, further investigations of major pharmacodynamic and pharmacokinetic studies were also suggested.

## 1. Introduction

**Figure 1:**
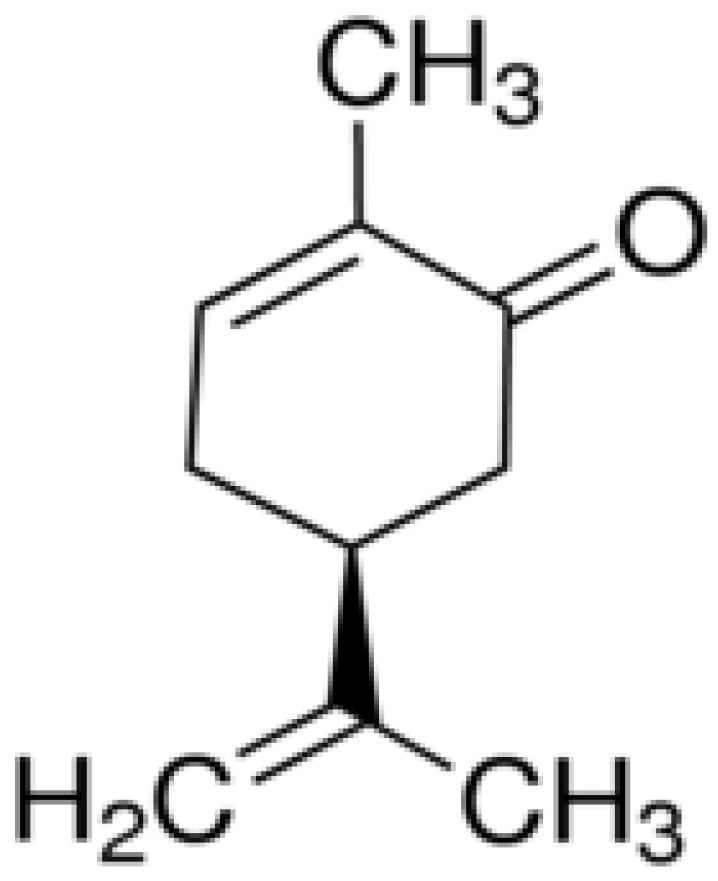
Chemical structure of carvone.

Carvone is a monoterpene ketone (2-methyl-5-(1-méthylethenyl)-2-cyclohexen-1-one) (C_10_H_14_O) (Figure 1) with a boiling point of 230 °C, which has an asymmetric carbon. Chemically, carvone exists in two forms (enantiomers (+)-carvone and (−)-carvone) with the same chemical and physical properties and which differ only in their rotatory power. This monoterpene is present in the essential oils of some plant species, including *Mentha* spp., *Origanum* spp., *Rosmarinus* spp., *Thymus* spp., and many others [1,2,3,4,5,6,7,8]. The concentration of this volatile compound differs according to the species and is related to parameters such as the species, the geographical location, the phenological stages, and the parts of the plant. It is synthetized and secreted as a secondary metabolite from essential oils, and its major role in the plant remains unclear. Currently, many studies have proven that carvone has promising pharmacological properties. Indeed, it has shown neuroprotective effects, and can therefore be developed as a drug against certain disorders such as depression, sedation, nociception, and seizure [9,10,11,12]. This molecule has also demonstrated an antidiabetic effect, through its role in the prevention of obesity and metabolic problems associated with high-fat diets, achieved by improving glycoprotein component abnormalities and controlling glucose metabolism [13,14,15]. The use of carvone as an antifungal has also been investigated against various fungi strains (*Candida* spp.), mycotoxigenic fungi (*Fusarium* spp., *Aspergillus* spp., and *Penicillium* spp.), and dermatophytes (*Trichophyton* spp., *Epidermophyton floccosum*, and *Microsporum* spp.) [16,17,18,19,20,21]. The action of carvone on germ tube formation and fungi biofilm was reported. Additionally, carvone can be used as an antibacterial agent against many strains of bacteria, including methicillin-resistant *Staphylococcus aureus* (MRSA) [20,22,23,24,25,26,27,28]. Its antibacterial effects are often related to its capacity to penetrate into bacterial cells thus inducing an increase in cell permeability and a decrease in cell membrane integrity. It also exhibited an antibiofilm effect against *S. aureus.* On the other hand, carvone had anticancer activity against different cancer cell lines, including myeloma and melanoma cells, and breast cancer cells [29,30,31,32]. The antiproliferative mechanism of action has also been elucidated. Moreover, some studies proved the anti-inflammatory property of carvone and investigated the underlying molecular mechanism [33,34,35,36]. Anticancer mechanisms of carvone are due to its different actions against checkpoints of cancer cells such as inducing apoptosis and cell cycle arrest. Other pharmacological activities have also been reported, including antioxidant activity [37], neuraminidase inhibitory activity against the influenza virus [38], and antiparasitic activity against *Culex quinque*, *Cx. pipiens*, *Aedes aegypti*, and *Haemonchus contortus* [39,40,41,42]. All these properties have enabled the use of carvone in other fields, such as the disinfection of food packaging and medical devices. In addition, to improve its action and extend its industrial use, carvone has been included in poly(lactic acid) films [16], antibacterial coating (ppCar) [23], and poly (lactic-co-glycolic acid) (PLGA) nanoparticles [25]. Therefore, the main objective of this paper is to report the pharmacological properties of carvone, and to highlight the mechanism(s) of action responsible for these activities.

## 2. Research Methodology

Literature data for all carvone studies was collected using different scientific search engines, including Scopus, Wiley Online, Web of Science, Scifnder, Google Scholar, PubMed, ScienceDirect, and SpringerLink. They were organized in tables and then analyzed, highlighted, and discussed. The chemical structure of carvone was drawn using ChemDraw Pro 8.0 software.

## 3. Results and Discussion

### 3.1. Natural Sources of Carvone

Carvone is the major compound of the essential oil (EO) of many species of the Lamiaceae family; *Mentha* [43,44,45,46], *Mentha spicata* [22,43,44,47,48,49,50,51,52,53,54,55,56,57,58,59,60], *Mentha × villoso-nervata* [43], *Mentha piperita* L. [48,53,61], *Mentha crispa* L. [61,62], and *Mentha cardiaca* L. [60]. Moreover, carvone is the major compound of the EO of *Anethum graveolens* [50,53,63,64,65,66,67,68], *Thymus vulgaris* [45], *Majorana hortensis* [45], *Carum carvi* [50,52,63,66,67,69,70,71,72,73], *Anethum sowa* [22], and *Solanum tuberosum* L. [74,75]. In addition, other species belonging to the Orchidaceae family have been characterized by their richness in carvone; *Catasetum discolor*, *Catasetum longifolium*, *Catasetum integerrimu*, *Catasetum macroglossu*, *Catasetum tabular*, *Catasetum veracruz*, *Catasetum viridiflavu* [76], and *Lippia alba* [18,28,77,78,79,80,81]. Secondary metabolites in EOs are variable depending on certain factors, including geographic origin, stages of development, and parts of the plant. Indeed, some studies have proven this variability between geographic locations and suggest the fluctuation of these phytochemicals in plants for responding to environmental situations [18,77,78].

### 3.2. Pharmacological Properties of Carvone

**Figure 2:**
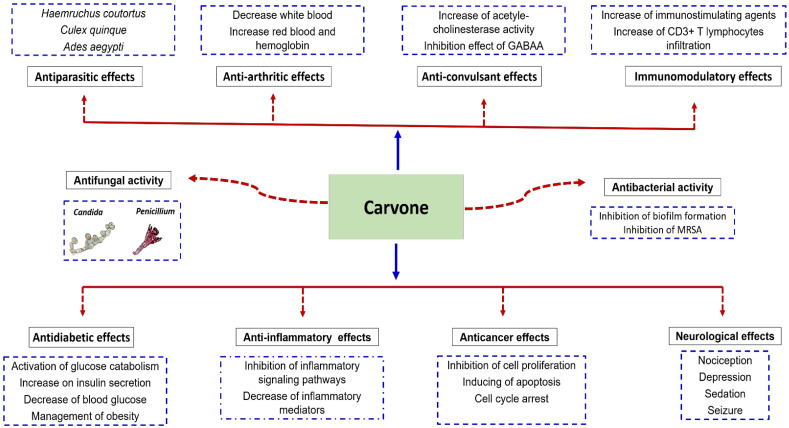
Pharmacological properties of carvones. Carvone exhibits multiple biological activities including antidiabetic, anti-inflammatory, anticancer, neurological, antimicrobial, antiparasitic, antiarthritic, anticonvulsant, and immunomodulatory effects.

With its varied actions on subcellular, cellular, and molecular actions, carvone exhibits several biological properties such as antimicrobial, anticancer, anti-inflammatory, antidiabetic, neurological, and many other pharmacological effects (Figure 2). 

#### 3.2.1. Neurological Activity 

Several authors have attempted to study the effect of the use of carvone on some neurological disorders such as depression, sedation, nociception, seizure, local anesthesia, as well as its effect on some receptors and on the action potential [9,10,11,12]. To study these effects, different models are used such as Swiss mice [9], Wistar rats [11], frog’s sciatic nerve [10], and cortical neurons prepared from the cerebral cortices of rat fetuses [12] (Table 1).

To assess the effect of carvone on disorders related to the central nervous system (depression, convulsion), De Sousa et al. [9] conducted their study using different enantiomers of carvone ((*S*)-(+)-carvone and (*R*)-(−)-carvone). The LD_50_ values of the enantiomers varied between 400–500 mg/kg, and both enantiomers demonstrated depressive effects, expressed as decreased ambulation and responsiveness to touch, as well as increased sedation, palpebral ptosis, and antinociceptive effects. In addition, (*S*)-(+)- and (*R*)-(−)-carvone reduce ambulation significantly. At 0.5 and 2.0 h after administration, (R)-(−)-carvone appeared to be more effective than its equivalent enantiomer [9]. However, at 1 h, (*S*)-(+)-carvone was slightly more powerful. To increase pentobarbital sleep duration, (*R*)-(−)-carvone (100 mg/kg) was more effective than (*S*)-(+)-carvone but was less effective at 200 mg/kg compared with its enantiomer, suggesting induction of a sedative effect. At a dose of 200 mg/kg, (*S*)-(+)-carvone substantially improved the latency of convulsions produced by PTZ and PIC, while (*R*)-(−)-carvone was ineffective against these convulsions. These findings indicate that (*S*)-(+)- and (*R*)-(−)-carvone have a CNS depressant effect, with an anticonvulsant property in (*S*)-(+)-carvone [9].

Faliagkas et al. [10] tested two enantiomers of carvone ((+)- and (−)-carvone) for their local anesthetic activity at two concentrations, 10 and 20 mM. The authors used a nerve preparation based on the frog’s sciatic nerve. They found that both enantiomers induce similar responses. When rinsed out of the nerve preparation and replaced with normal saline, they completely abolished the action potential of the evoked compound within 6–7 min, with an instantaneous recovery of 83–87%. Both carvones acted in the same way as 10 mM lidocaine (a standard local anesthetic), although they were 3–4 times less active in terms of reaction time. There was no recovery of the elicited compound action potential when the nerves were exposed to carvones for more than 6–7 min, suggesting a neurotoxic effect. In conclusion, the unique neurotoxic action of (+)- and (−)-carvone may be a disadvantage in therapeutic practice.

Based on multiple previous reports regarding the insecticidal activity of carvone and its effects on the nervous system, Sánchez-Borzone et al. [12] studied the effect of both carvone enantiomers on the GABAA receptor as a major insecticidal target by determining their effects on the recognition sites of benzodiazepines (BZD), belonging to the group of sedatives and anxiolytics, using primary neuronal cultures. Both isomers were able to block GABA-induced stimulation of (^3^H) flunitrazepam binding, suggesting that they act as negative allosteric modulators on the GABAA receptor. Their action was equivalent to that of thujone in this study, with the (*R*)-(−)- carvone stereoisomer being the most potent. The unusual configuration of the isopropenyl group at position five appears to be important for receptor engagement, whereas the structure of carvone does not appear to be important for receptor recognition. In a mouse neuron culture system, the doses required to produce negative receptor modulation were not lethal. These findings support the theory that carvones’ insecticidal effect is explained, at least in part, by their interaction with the non-competitive blocker site of the GABAA receptor.

Gonçalves et al. [11] previously demonstrated that the in vivo antinociceptive activity of (−)-carvone is impaired by a decrease in nerve excitability. In their study, they attempted to investigate and reveal the neuropharmacological effect of carvone to explain the observed effect (compound action potential (CAP) inhibitory effect) [11]. Using a modified single sucrose-gap technique (ex vivo), the effects of (+)- and (−)-carvone on CAP properties were evaluated. The study findings showed that (−)-carvone was less potent (IC_50_ = 10.70.07 mM) in reducing nerve excitability than its enantiomer, (+)-carvone (IC_50_ = 8.70.1 mM), despite having a similar mode of action, as their effects were partially counteracted by nerve washing and also by a reduction in depolarization velocity, most likely due to voltage-gated sodium channel blockades. These findings suggest that monoterpene suppression of CAP conduction in peripheral nerves may further enhance knowledge about the pharmacology of natural bioactive substances. Furthermore, changing the chemical structures of such molecules may be used to activate or inhibit neuronal excitability [11].

#### 3.2.2. Antidiabetic Activity

Several studies showed the antidiabetic effects of volatile compounds including carvone [82]. Indeed, three separate studies were interested in the potential antidiabetic activity of carvone by revealing its overall activity on in vivo models such as C57BL/6 mice with high-fat diet-induced obesity and streptozotocin (STZ)-induced diabetes in Wistar rats. Those studies have also focused on the main underlying mechanism of action exhibited by this molecule following multiple biochemical, hematological, and histopathological analyses [13,14,15] (Table 2).

To verify whether S-carvone can prevent obesity and metabolic problems caused by a high-fat diet, Alsanea and Liu [13] conducted a study on ten-week-old C57BL/6 male mice fed a high-fat diet and injected, intraperitoneally twice a week, with benzyl isothiocyanate (BITC), S-carvone, or vehicle for 8 weeks. Body weight, food consumption, and body composition were all monitored, and glucose tolerance and insulin tolerance tests were performed at the end of the study. Moreover, to determine the effects of BITC and (*S*)-carvone therapies on lipid and glucose metabolism and inflammatory responses, serum biochemistry, histology, and gene expression analyses were carried out. Therefore, (*S*)-carvone and BITC inhibited the weight gain induced by a high fat diet, as well as the insulin resistance and the accumulation of fat in the liver. The positive effects were related to increased expression of macrophage marker genes in white adipose tissue, including *F4/80*, *Cd11b*, *Cd11c*, *Cd206*, and *Tnf-α*, and decreased expression of genes involved in production and transport of lipids in the liver (*Ppar2*, *Scd1*, and *Cd36*). In conclusion, this study suggests that BITC and (*S*)-carvone block high-fat diet-induced obesity and metabolic disorders and may be considered for the management of the obesity epidemic [13].

Muruganathan et al. [14] conducted their research to examine the impact of carvone on glycoprotein disruption in the STZ-induced diabetes model. A single intraperitoneal dose of STZ (40 mg/kg b.w.) induced diabetes in male Wistar rats. Glycoprotein levels were altered in experimental diabetes mellitus. Carvone was administered intragastrically to diabetic rats at doses of 25 mg/kg, 50 mg/kg, and 100 mg/kg for 30 days. Carvone’s effects on plasma glucose, insulin, plasma, and tissue glycoproteins were evaluated. In experimentally diabetic rats, oral treatment with carvone (50 mg/kg b.w.) for 30 days improved glycemic status in a dose-dependent manner, with a substantial increase in plasma insulin levels, and decrease in plasma glucose levels. The abnormal levels of plasma and tissue glycoprotein components were nearly normalized. Current results indicate that carvone, in addition to its antihyperglycemic action, may be able to improve glycoprotein component abnormalities in experimental diabetes. In view of these encouraging results, it is recommended to expand the scope of carvone usage in further studies to mitigate the negative consequences of diabetes [14].

Muruganathan et al. [14] investigated the impact of carvone on carbohydrate metabolic enzymes in the livers of normal and STZ-induced diabetic rats. A single intraperitoneal dose of STZ (40 mg/kg b.w.) was used to induce diabetes. STZ injection caused a substantial increase in plasma glucose and glycosylated hemoglobin (HbA1c), as well as a reduction in insulin and hemoglobin (Hb) levels. Carbohydrate metabolic enzymes, glycogen, enzymatic antioxidants in the pancreas, and hepatic marker levels have all been affected. Diabetic rats treated daily with a single oral dose of carvone (50 mg/kg b.w.) for 30 days, showed a substantial decrease in plasma glucose and HbA1c levels, as well as a significant improvement in Hb and insulin levels [14]. Administration of carvone restored the reversed activity of carbohydrate metabolic enzymes, enzymic antioxidants, and hepatic marker enzymes in diabetic rats to near-normal levels. The results were compared with gliclazide, a common oral hypoglycemic drug. Histopathological examination of the liver and pancreas, as well as immunohistochemistry of the pancreas, showed that carvone therapy decreases STZ-induced damage to liver and pancreatic cells. According to these findings, carvone controls glucose metabolism by improving enzymes important in the hepatic tissues of diabetic rats. Nevertheless, more research and safety studies are required to further verify carvone’s benefits [14].

#### 3.2.3. Antifungal Activity 

Carvone has emerged as a promising antifungal compound. Its application extends from the screening of basic properties against different fungi strains and mycotoxins, to an application designed in the food industry, in particular food packaging [16,17,18,19,20,21] (Table 3). 

In their ultimate goal to develop antifungal poly(lactic acid) (PLA) films for food packaging applications, Boonruang and collaborators [16] used (*R*)-(−)-carvone in their study. The molecule was incorporated into PLA-based polymer at 10%, 15%, and 20% by weight. The film conversion process consists of three steps, namely, melt blending, sheet extrusion, and biaxial stretching. The incorporation of antifungal compounds into the polymer matrix resulted in decreased Tg and Tm, increased gas permeability, reduced tensile strength, and increased elongation at the break of the antifungal PLA films. The antifungal films were homogeneous and transparent.

Giovana et al. [17] were interested in finding an effective antifungal drug in the fight against candidiasis, an infection caused by *Candida* spp. which has developed significant resistance to current therapies. Since it has already been documented that *Mentha* spp. has antifungal properties, the authors of this research chose four main components present in its EO, including carvone. They evaluated growth suppression by microdilution, biofilm breakdown by electron microscopy, and germ tube formation inhibition by optical microscopy. The compounds tested had an antifungal activity with a MIC of 0.5 mg/mL, at least 50% biofilm inhibition at the 0.5 mg/mL concentration, polymorphism inhibition at 86%, and changes in the cell envelope of yeast (SEM) and cell viability greater than 50% among the *Candida* strains tested. Due to the potential antifungal capacity of carvone, as well as its low cytotoxicity, it was considered a viable candidate to supplement antifungal regimens.

Mesa-Arango et al. [18] selected two carvone chemotypes from Colombian *L. alba* (Mill.) EOs to investigate their antifungal activity against multiple strains such as *Candida parapsilosis*, *Candida krusei*, *Aspergillus flavus*, and *Aspergillus fumigatus* strains using standardized protocols. According to the research results, the GM-MIC values were greater than 500 g/mL against the various strains tested, suggesting a weak antifungal activity.

In their study, Morcia et al. [19] selected certain natural EO compounds (including carvone) on 10 species of mycotoxigenic fungi involved in several plant diseases, namely, *Fusarium subglutinans*, *Fusarium cerealis*, *Fusarium verticillioides*, *Fusarium proliferatum*, *Fusarium oxysporum*, *Fusarium sporotrichioides*, *Aspergillus tubingensis*, and *Aspergillus carbonarius*. Carvone and the other chemicals examined had a toxic effect on mycelium development in vitro on all fungal species, albeit at varying levels of activity, prompting additional research on these compounds in the field of mycotoxins.

In a recent study, Piras et al. [21] examined the antifungal efficacy of *Mentha spicata* L. EO, containing 62.9% carvone. Their primary objective was to test its effectiveness on the virulence factors of *Candida albicans*, especially the suppression of germ tube development, as well as their impact on other strains. Consequently, *M. spicata* EO showed a superior effect against *Cryptococcus neoformans* and the dermatophytes *Trichophyton rubrum* and *Trichophyton verrucosum* (0.32 μL/mL) and also inhibited germ tube formation in *Candida albicans* up to 80% at concentrations eight times lower than the MIC. The results of the study support and validate the use of this plant EO in traditional medicine.

Given the reported activity of carvone in the literature, Moro et al. [20] conducted a research to assess the antifungal activities of (+)- and (−)-carvone, (+)- and (−)-hydroxydihydrocarvone, and α,β-epoxycarvone. (+)-Hydroxydihydrocarvone (HC+), (−)-Hydroxydihydrocarvone (HC−), and, α,β-epoxycarvone (EP) were synthesized from (+)-carvone (C+) or (−)-carvone (C−). The antifungal activity (MIC and MFC) was tested against *Candida parapsilosis*, *Candida tropicalis*, *Candida krusei*, and *Candida albicans*. All compounds showed modest antifungal efficacy against *Candida tropicalis* and *Candida parapsilosis*. Moreover, EP and C+ had modest antifungal activity against *C. krusei*. The results indicate that carvones and their derivatives may be used as antifungal drugs against *Candida* yeasts.

#### 3.2.4. Antibacterial Activity

The antibacterial activity of carvone has been studied by several authors against multiple strains such as *Escherichia coli*, *S. aureus*, *Streptococcus faecalis*, and *Pseudomonas aeruginosa*. Different enantiomers were used in comparative studies to assess the structure–function relationship. Some studies were interested in the antibacterial effect of the encapsulated form (carvone loaded PLGA nanoparticles), others in its microbial transformation, while others were interested in manufacture of carvone biofilms (antibacterial coating) to prevent bacterial colonization of medical devices [20,22,23,24,25,26,27,28] (Table 4).

Aggarwal et al. [22] investigated the antibacterial effects of *Mentha spicata* (containing (*S*)-carvone as main component (56%) and *Anethum sowa* Roxb. (containing (*R*)-carvone as major component (50.4%)). Evaluation of the in vitro bioactivity of the separated oily components showed that both optical isomers of carvone were active against a wide range of microorganisms tested. The activity of these monoterpene enantiomers was found to be similar to the bioactivity of the oils in which they were discovered.

Since current coating techniques, such as immobilization of antimicrobial compounds, time-releasing antibiotic agents, and silver nanoparticles, require multiple processing steps and have low efficacy and stability, Chan et al. [23] proposed a single-step plasma polymerization of carvone to produce a moderately hydrophobic antibacterial coating (ppCar) with an average roughness < 1 nm. Even after 10 days of air aging, ppCar maintained a static water contact angle of 78° and remained stable for 24 h in LB broth immersion. ppCar performed well in the live/dead fluorescence test and the crystal violet assay. The biofilm test effectively reduced *E. coli* (86%) and *S. aureus* (84%) bacteria. For its bactericidal actions, it has also been shown that ppCar perforates the bacterium membrane. The cytotoxicity test revealed that the coating is not harmful to human cells. This work would be of interest to researchers interested in creating a bacteria-resistant and biocompatible coating on various substrates at low cost.

In a novel research, the microbial transformation of C- was investigated (metabolized by the phytopathogenic fungus *Absidia glauca*) by Demirci et al. [24]. The diol 10-hydroxy-(+)-neodihydrocarveol was produced after 4 days of incubation. X-ray diffraction and spectroscopy methods were used to determine the absolute arrangement and structure of the crystalline material (MS, IR, and NMR). Human pathogenic bacteria were used to test the antimicrobial activity of the substrate and metabolite. The main results of the study showed that the inhibitory action of the metabolite is modest.

In their research, Fatondji et al. [26] synthesized semicarbazone and thiosemicarbazone from R-(−)-carvone via direct condensation of semicarbazide or thiosemicarbazide in an acidic medium and evaluated their antibacterial activity. The purity of the synthesis products was determined by thin layer chromatography (TLC) after recrystallization, and their structures were verified by IR spectroscopy, nuclear magnetic resonance (NMR), and mass spectrometry (MS). The compounds were evaluated on *S. aureus*, *S. faecalis*, *P. aeruginosa*, and *E. coli* strains. Therefore, the chemicals inhibited *P. aeruginosa* growth, with MIC values of 78.1 and 312.5 μg/mL for thiosemicarbazone and semicarbazone, respectively. With a MIC of 39 μg/mL, thiosemicarbazone was also active against *S. aureus*. On the other hand, *P. aeruginosa* and *S. aureus* have become increasingly resistant to antibiotics such as oxacilline (MIC = 1.5 μg/mL for *S. aureus*) and cefixime (MIC < 1 μg/mL). Both compounds showed poor antibacterial activity against *E. coli* and *E. faecalis.* Furthermore, the thiosemicarbazone showed intriguing action against *S. aureus* and *P. aeruginosa.*

In order to extend the antibacterial action of carvone, Esfandyari-Manesh et al. [25] attempted to create poly (lactic-co-glycolic acid) (PLGA) nanoparticles. These nanoparticles were created using emulsification solvent evaporation (ESE) and nanoprecipitation techniques. Nanoparticles were studied for their shape, size and size distribution, drug loading, entrapment efficiency, release profile, and antibacterial efficacy. This allowed to generate nanoparticles with smaller size (126 nm), narrower size distribution (PDI of 0.08–0.2), higher drug loading (12.32 percent carvone), as well as better microbial growth suppression than ESE. Carvone drug release experiments in vitro at 37 °C for 4 days revealed an early burst (36%). The MIC of carvone-loaded nanoparticles against *S. aureus* and *E. coli* was 182 and 374 mg/mL, respectively. The nanoparticles created in this research were of the appropriate size and shape, and according to the antimicrobial research, EO-loaded PLGA nanoparticles may be useful in medicinal and culinary applications.

Given the reported activity of carvone in the literature, Moro et al. [20] conducted research to assess the antibacterial activity (MIC and MBC) of C+, C−, HC+, HC−, and EP against *E. coli* and *S. aureus*. In addition, HC+, HC−, and EP have been synthesized from C+ or C-. The results showed that C− and HC− possessed a weak antibacterial action against *E. coli*. In contrast, EP, C+, and HC+ had no effect on the bacterial strains examined.

The aim of Mun et al.’s [27] study was to evaluate the antibacterial activity of R- and S-carvone in combination with gentamicin (GET) against MRSA. The latter is a gram-positive bacterium which causes nosocomial pneumonia, abscesses, and surgical site infections. Multidrug resistance is common in nosocomial MRSA infections. The broth micro-dilution method was used in this research to evaluate the antimicrobial sensitivity of R- and S-carvone and GET. The MIC for R- and S-carvone against six distinct strains of *S. aureus* ranged from 500 to 1000 µg/mL. To explore the possible synergistic effects of various combinations of carvone enantiomers and GET, anti-MRSA activity was assessed using the checkerboard and time-kill tests. The results determined that R-carvone in combination with S-carvone, R-carvone in combination with GET, and S-carvone in combination with GET all showed substantial synergistic efficacy against MRSA. These results imply that the combination treatment successfully increases the anti-MRSA monotherapy activities of R-carvone, S-carvone, and GET. Carvone has been shown to be a possible adjuvant antibacterial agent in this research.

*L. alba* EO and its main components (citral and carvone) were tested in vitro by Porfírio et al. [28] for their antibacterial and antibiofilm properties against *S. aureus*. Hydrodistillation was used to extract the EOs from *L. alba* aerial parts, which were then evaluated by GC-MS. The microdilution method was used to determine the MIC and MBC. The biomass development in the biofilm was assessed using the microtiter-plate method with the crystal violet test for the antibiofilm assays, and the viability of the bacterial cells was examined. The essential oil and its main component (carvone) have shown antibacterial action. At a dosage of 0.5 mg/mL, there was 100% suppression of *S. aureus* biofilm formation. However, at doses of 0.5 to 2 mg/mL, eradication of biofilm cells has been verified. The results of the present study indicate the antibacterial and antibiofilm capacity of *L. alba* EO against *S. aureus*, a species of known therapeutic relevance.

#### 3.2.5. Antibacterial and Antibiofilm Activities

Regarding the promising antibiofilm activities of carvone and citral against *S. aureus* bacteria, an in vitro study was carried out in this regard by Porfírio et al. [28] on the EO of *L. alba* and its main components (citral and carvone). Three EOs (LA1EO, LA2EO, and LA3EO) were extracted from the aerial parts of three specimens of *L. alba* by hydrodistillation and analyzed by GC-MS. MIC and MBC values were determined by the microdilution method. Regarding the assay of the formation of biomass in the biofilm, it was evaluated by the microtiter plate technique with the assay of crystal violet and the analysis of the viability of bacterial cells. The results of the present research suggest that all of the oils and their major components have antibacterial activity, and the lowest MIC and MBC values were 0.5 mg/mL when LA1EO and citral were used. Likewise, a potential 100% inhibition of *S. aureus* biofilm formation was observed at the concentration of 0.5 mg/mL of all EOs. In contrast, the elimination of cells from the biofilm was confirmed at concentrations of 1, 2, 2, and 0.5 mg/mL for LA1EO, LA2EO, LA3EO, and citral, respectively.

#### 3.2.6. Antiviral Activity

Carvone was also tested for its antiviral effects (Table 5).

Given the recent outbreaks of highly dangerous influenza viruses, it was found that it is imperative to develop new anti-influenza drugs. In their *in-silico* study, they designed 36 ligands to analyze how they bind to neuraminidase (NA) active sites. The design is based on structural resemblance to the commercial inhibitor, oseltamivir (OTV), ligand polarity, and amino acid residues in the NA active sites. Their study result suggests that one of the designed ligands had the lowest binding energy (∆G_bind_) (−8.30 kcal/mol), comparable with OTV (−8.72 kcal/mol), with seven hydrogen bonds formed. Since the stability analysis indicated that the A18-NA complex was stable, this study encourages further research to synthesize and evaluate this compound [38].

#### 3.2.7. Antioxidant Activity

Oxidative stress is often implicated in several severe and chronic illnesses such as cancers, cardiovascular diseases, diabetes, and many others. Since antioxidant compounds can mitigate oxidative stress due to of their antiradical ability and/or reducing power, the search for new effective and safe antioxidants from plants has intensified in recent years [83]. As part of this research, carvone has been investigated for its antioxidant effect by various researchers. One of the first investigations on the antioxidant properties of carvone isolated from *Mentha spicata* was carried out by Elmastaş et al. [55]. The results of the total antioxidant activity test indicated that S-carvone possess high antioxidant activity compared with α-tocopherol, used as a reference antioxidant.

Carvone was investigated for its antioxidant potential by various in vitro systems, including lipid peroxidation, 2,2-dipenyl-1-picrylhydrazyl (DPPH), and phosphomolybdenum assay [37]. In this study, carvone isolated from *Z. alatum* showed inhibitory activity against thiobarbituric acid reactive species (TBARS) induced by some pro-oxidants (10 µM FeSO_4_ and 5 µM sodium nitroprusside) in rat liver and brain homogenates. Carvone also caused the scavenging of the DPPH radical and the reduction of molybdenum, Mo(VI) to Mo(V). Galstyan et al. [84] documented the antioxidant property of a synthesis of carvone-derived 1,2,3-triazoles. The conjugates prepared demonstrated high antioxidant activity.

#### 3.2.8. Anti-Inflammatory Activity

The anti-inflammatory effects of terpenoids compounds such as carvone were revealed by numerous studies [85]. Monoterpene α,β-epoxy-carvone was evaluated by da Rocha et al. [33] for its anti-inflammatory properties. This was carried out using the acetic-acid-induced peritoneal capillary permeability test. The results showed that the intraperitoneal administration of α,β-epoxy-carvone (300 mg/kg) inhibits the increase in vascular permeability caused by acetic acid. These findings suggest that α,β-epoxy-carvone inhibits the acute inflammatory reaction.

The anti-inflammatory activity of cyane–carvone (CC), a monocyclic monoterpene, was evaluated by the methods of bradykinin, histamine, prostaglandin E_2_ (PGE_2_), serotonin, and carrageenan-induced paw edema in mice [34]. It was found that in bradykinin, histamine, PGE_2_, and serotonin tests, 75 mg/kg CC significantly decreased paw edema (t = 30, 60, 90, and/or 120 min). While in the carrageenan test, 50 mg/kg and 75 mg/kg CC (t = 3 h and t = 4 h) and 25 mg/kg CC (t = 4 h) significantly decreased paw edema.

Zhao and Du [35] examined the anti-inflammatory and defensive role of _D_-carvone on lipopolysaccharide (LPS)-initiated lung damage in mice. As a result, _D_-carvone significantly attenuated the lung damage produced by LPS by reducing the lung wet-to-dry (W/D) ratio as well as the amount of total cells, macrophages, and neutrophils in BALF. An important reduction in serum TNF-α, IL-1β, and IL-6 levels was observed in _D_-carvone treated mice. This molecule also altered histopathological disorders due to LPS-initiated lung damage. The effects of _D_-carvone were comparable with those of the positive control, dexamethasone. This study indicates that pre-treatment with _D_-carvone significantly provided an anti-inflammatory and protective effect against LPS-instigated lung damage.

The molecular mechanism responsible for the anti-inflammatory properties of (*S*)-(+)-carvone has been highlighted by Sousa et al. [36]. The results of this study show that (S)-(+)-carvone is a novel Sirtuin-1 (SIRT1) activator with the potential to counteract the chronic low-grade inflammation characteristic of age-related diseases.

This substance appears to have various uses, this time following a study by da Rocha et al. [33] on mice, to examine the antinociceptive and anti-inflammatory activities of α and β Epoxy-carvone monomers, extracted from EOs of many plant species or obtained by organic synthesis. After intraperitoneal administration of this monomer at doses of 100 mg/kg, 200 mg/kg, or 300 mg/kg, a significant antinociceptive effect was observed, as shown by the abdominal contortion test induced by acetic acid, with a decrease in blood pressure. Nociception was induced by formalin in the first (300 mg/kg) and second phase (200 and 300 mg/kg). These results suggest that the α and β Epoxy-carvone monomer inhibits the acute inflammatory response, with a peripheral and central antinociceptive effect observed in mice which can be explained by the activation of the opioid system, responsible for the antinociceptive activity induced by this monomer.

Another work was carried out by Mogosan et al. [86] in order to have a qualitative and quantitative comparative analysis of the chemical composition and to evaluate the anti-inflammatory and antinociceptive effects of the EOs of three species of Mentha (*Mentha piperita* L. var. pallescens (white peppermint), *Mentha spicata* L. subsp. crispata (spearmint), and *Mentha suaveolens* Ehrh. (pineapple mint)) grown in Romania. The anti-inflammatory activities of EOs were determined by the carrageenan-induced rat paw edema test, while the antinociceptive activity was assessed by the contortion test in mice, using a solution of 1% (*v*/*v*) acetic acid administered intraperitoneally and by the hot plate test in mice. The data from this study showed that the EOs chemotype of *M. spicata* L. (EOMSP) produced statistically significant and dose-dependent anti-inflammatory and antinociceptive effects.

#### 3.2.9. Anticancer Activity

**Figure 3:**
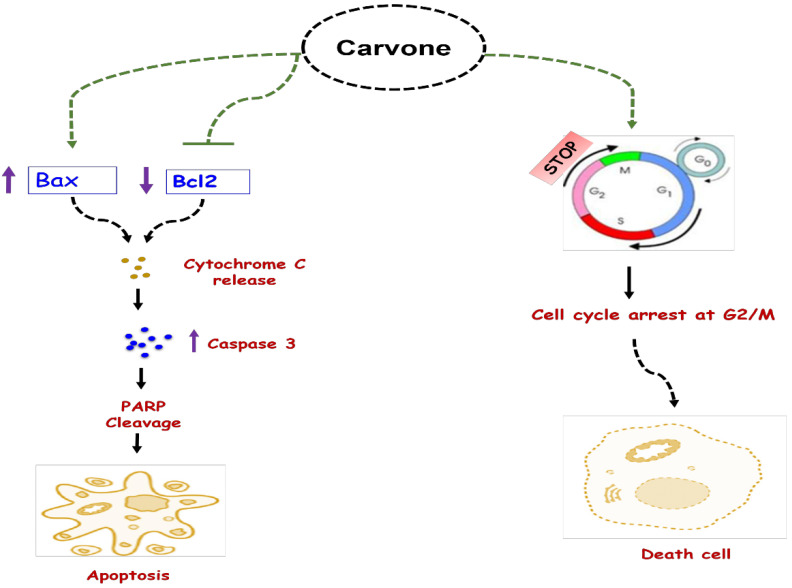
Anticancer mechanisms of carvone. Carvone can induce anticancer effects by two main mechanisms: (1) intrinsic apoptotic action via decreasing Bcl2 and decreasing Bax, as well the release of cytochrome C which induce caspases expression and PARP cleavage; (2) cell cycle arrest at G_2_/M via its action on cyclin-dependent kinase 1.

Similar to several other bioactives which showed promising anticancer properties [87,88,89,90,91,92,93], carvone has also been studied for its anticancer properties. Indeed, several in vitro investigations based on cell culture tests showed that this compound exhibits antiproliferative effects against various cancer cell lines. In this sense, the results of the study conducted by Ding et al. [29] demonstrated that carvone exerts significant antiproliferative effects against myeloma cancer cells in a dose-dependent manner. The anticancer activities were linked to the induction of apoptosis and the G_2_/M cell cycle arrest (Figure 3). Moreover, carvone could inhibit cell invasion and p-P38 protein expression at an IC_50_ of 20 μM. In another study, Gopalakrishnan et al. [30] evaluated the chemopreventive efficacy of _D_-carvone (at 10, 20, and 30 mg/kg b.w.) in vivo using 7,12-dimethylbenz(a)anthracene (DMBA)-induced skin carcinogenesis. The results showed that the tumor incidence of 100% in DMBA-painted animals as well as _D_-carvone at a dose of 20 mg significantly prevented skin carcinogenesis. In addition, this study showed decreased levels of phase I enzymes (cytochrome P450 and cytochrome b5) with increased levels of phase II enzymes (GR, GST, and GSH) and increased expression of Bax, caspase-3, and caspase-9 with decreased expression of mutated p53 and Bcl-2 in animals treated with DMBA and _D_-carvone (20 mg).

In another experiment [31], carvone was investigated for its ability to reduce melanin content in melanoma cells as well as for its beneficial effects mediated by the cAMP pathway. This study found that carvone decreases the melanin content and inhibits melanoma cell proliferation in a dose-dependent manner. Likewise, it caused the inactivation of cell cycle-associated proteins such as cyclin-dependent kinase 1 (CDK1). It should be noted that the beneficial activities of carvone were abrogated by cAMP inhibition.

Furthermore, Patel and Thakar [32] evaluated the antiproliferative and apoptotic activity of L-carvone, and the underlying mechanism(s) of action on breast cancer (MCF 7 and MDAMB 231) and normal (MCF 10A) cell lines. Results showed that L-carvone exhibited a strong antiproliferative effect against MCF7 (IC_50_ = 1.2 mM) and MDA MB 231 cells (IC_50_ = 1.0 mM), inhibited the migration of breast cancer cell lines, and induced apoptosis. L-carvone exposure arrested MCF 7 cells in S phase of the cell cycle and caused DNA damage that was apparent from the increased tail movement, which could be caused by an increase in reactive oxygen species (ROS). Moreover, the glutathione levels were increased. Finally, p53 and caspase-mediated apoptosis was attributed to the increased level of p53, Bad, cleaved caspase 3, and cleaved PARP (Figure 3).

#### 3.2.10. Antiparasitic Activity

Parasitic diseases are infectious diseases caused by parasites which, under favorable conditions, can be transmitted to humans [94], among them African trypanosomiasis, malaria transmitted by Plasmodium, schistosomiasis, and leishmaniasis. Parasitic diseases are still responsible for many health problems. These parasites are responsible for a high rate of mortality and morbidity each year in endemic countries [95], and are probably responsible for more than 1 to 2 billion infections, which result in several million deaths each year [96].

Current chemotherapy is based on developed synthetic drugs [97]. However, unfortunately, a number of these chemotherapeutic drugs against parasites have serious side effects [98]. In addition, some parasites develop resistance to the treatments [99]. The majority of people who suffer from these parasitoses often live in developing countries and have only low incomes. It is therefore urgent to discover alternative treatments against these diseases. Numerous studies have elucidated the role of plants against anthelmintic in traditional medicine [100,101,102]. However, a wide range of molecules isolated and identified from plants have shown promising activity against multiple parasites [103,104,105,106].

A limited number of studies have reported the parasitic activity of carvone [39,42]. Pavela, [39] evaluated the efficacy of carvone and other aromatic compounds and their mutual binary combinations for acute toxicity against *Culex quinquefasciatus* larvae. The results show that carvone is one of the substances identified with the highest synergistic effect on larval mortality. Although these results are more important when combining two aromatic molecules, in this context, among the combinations that show a higher synergistic effect on larval mortality there are carvone and carvacrol, carvone and 4-allylanisole, carvone and α-terpineol, and finally carvone and menthone [39].

The evaluation of larvicidal activity of *Mentha* species EOs and their isolated major components against the West Nile virus mosquito (*Cx. pipiens* 3rd–4th instar larvae) showed that *Mentha pulegium* EO and its major components have an important anti-mosquito activity. Carvone showed moderate larvicidal activity against *Cx. pipiens* larvae of biotype molestus, with LC_50_ (95% CL) and LC_90_ (95% CL) values of 84.58–106.77 and 137.02–228.97, respectively [40]. In another study, Lima et al. [42] evaluated the larvicidal activity of *Mentha x villosa* EO (MVEO) and its major constituent, rotundifolone, against larvae of *Aedes aegypti*. The results of the study showed that MVEO exhibited outstanding toxic effects against *Ae*. *aegypti* larvae (LC_50_ = 45.0 ppm). The results of the comparative study between rotundifolone and the molecules isolated from the plant showed that all tested compounds were less potent than rotundifolone (LC_50_ = 62.5 ppm), except D-limonene. Concerning carvone, it exhibited intermediate larvicidal activity with (+)-carvone epoxide (LC_50_ = 254.6 ppm) and (−)-carvone epoxide (LC_50_ = 217.5 ppm) [42].

Katiki et al. [41] assessed in vitro the anthelmintic activity of aromatic compounds present in plants, on eggs collected from sheep droppings infected with the multidrug-resistant strain of gastrointestinal nematode *Haemonchus contortus*. The results obtained show that carvone is one the five promising compounds with anthelmintic activity with an LC_50_ value of 0.085 mg/mL [41].

#### 3.2.11. Anti-Arthritic Activity

In a study conducted on arthritic rats, Chen et al. [107] evaluated the anti-arthritic activity of _D_-carvone against arthritis induced by Freund’s complete adjuvant (FCA) in rats. Following oral administration of _D_-carvone for 25 days at doses of 30 mg/kg and 60 mg/kg against FCA-induced arthritic rats, certain changes were observed, namely, improvement in body weight, reduction in leg swelling, edema formation, and organ index in these arthritic rats. Other improvements were also recorded concerning the decrease in the levels of white blood cells, with an improvement in the levels of red blood cells and hemoglobin, a decrease in the levels of lipid peroxidation, with the observation of a significant increase in the levels of enzymatic and non-enzymatic antioxidants. The results of this study showed the crucial role of _D_-carvone in significantly modulating inflammatory cytokine levels and improving ankle joint pathology against FCA-induced arthritic inflammation implying significant antiarthritic activity in rats.

#### 3.2.12. Anticonvulsant Activity

Many drugs block seizures, but with little effect in preventing or curing this disease. Thus, the pharmaceutical industries continue to develop new safe and effective therapeutic alternatives to the management of epilepsy. In this perspective, and in a study conducted by Costa et al. [108] in a model of epilepsy induced by pilocarpine, CC showed an anticonvulsant effect. Indeed, administration of the 25 mg/kg, 50 mg/kg, or 75 mg/kg doses of CC resulted in a reduction of 16.7%, 33%, and 66.7%, respectively, against pilocarpine-induced seizures, and was effective to increase both the latency to first seizures and the percent survival, resulting in 33.3%, 67%, and 91.7% protection against seizure-induced death, respectively. Knowing that the reference drug atropine administered at a dose of 25 mg/kg creates significant 100% protection, likewise, the results of this study were marked by an increase in acetylcholinesterase activity in the hippocampus of mice after seizures induced by pilocarpine. These data suggest the clear association of the anticonvulsant capacity of CC and the increased activity of the enzyme acetylcholinesterase.

In addition, another study was conducted on models of epilepsy by Marques et al. [109] to study the effects of CC against seizures induced by pilocarpine (PILO), pentylenetetrazole (PTZ), and picrotoxin (PTX) in mice. After acute CC treatment at repeated oral doses (25 mg/kg, 50 mg/kg, and 75 mg/kg) for 14 days, positive anticonvulsant effects in PILO, PTZ, and PTX epilepsy models were recorded. Furthermore, it was shown that this substance could act on an allosteric site of GABAA, different from the site of action of BZD.

#### 3.2.13. Anxiolytic Activity

Another research work was performed on male Wistar rats by Hatano et al. [78], this time showing the anxiolytic effects of (*R*)-(−)-carvone, extracted from *L. alba* EO which is widely used in the regions of Central and South America as a tranquilizer.

Data from this research suggests that *L. alba* may exert anxiolytic-like effects on a specific subset of defensive behaviors that have been implicated in generalized anxiety disorder, and suggest that carvone is one of the constituents of *L. alba* responsible for its action as a tranquilizer.

#### 3.2.14. Immunomodulatory Activity

On mice, another study was carried out by Lasarte-Ciae al. [110] in order to explore the immunomodulatory capacity of a series of compounds representing each of the 10 categories or groups of odors, including that of carvone, while highlighting its potential as a therapeutic agent for diseases related to the CNS. During this research, the impact of each particular odor on the immune response was evaluated after immunization with the model ovalbumin antigen in combination with the TLR_3_ agonist poly I:C. As a result of this study, it was shown that some odors can behave as immunostimulating agents, while others could be considered as potential immunosuppressive odors. In this context, it was found that inhaling the odor of carvone can have an immunostimulating effect with improved memory capacity in BALB/c and immunosuppressive mice, consequently leading to a deterioration of the memory ability in C57BL/6J mice, while facilitating or altering viral clearance, respectively, in a model of viral infection with a recombinant adenovirus. This can be explained by a higher infiltration of CD3^+^ T lymphocytes in the hippocampus and an increased local expression of the mRNA encoding the cytokines IL-1β, TNF-α, and IL-6, and a decrease in the number of CD3 and an increase in IFN-γ.

#### 3.2.15. Antispasmodic Activity

Another intestinal antispasmodic virtue of C- has been revealed in a study conducted by Souza et al. [111]. The terminal parts of the ileum were mounted for isotonic contraction recordings. The effect of C− has been compared with that of verapamil, which is known to be a classic calcium channel blocker. The results of the measurement of the contractile response caused by C- showed that this monoterpene reduces the contraction induced by a high K+, with 100 times more potency than verapamil. This is a typical action of the classic type of calcium channel blocker.

#### 3.2.16. Acaricidal Activity

In 2015, Peixoto et al. [77] demonstrated the acaricidal activity of EOs of *L. alba* genotypes and of its main monoterpenes (carvone, limonene, and citral) against *Rhipicephalus microplus* ticks. The data obtained showed that citral had the greatest efficacy against *R. microplus* larvae, with an LC_50_ of 7.0 mg/mL, followed by R-(−)-carvone (LC_50_ = 9.9 mg/mL), and S-(+)-carvone (LC_50_ = 10.9 mg/mL). However, the limonene enantiomers reached LC_50_ values of 31.2 mg/mL for R-(+)-limonene and 54.5 mg/mL for S-(−)-limonene. In this regard, these results suggest that carvone as a major component of this EO may constitute an ecological alternative in the control of ticks in livestock.

#### 3.2.17. Antimanic Activity

In a study by Nogoceke, et al. [112], the antimanic activity of (*R*)-(−)-carvone and (*S*)-(+)-carvone was evaluated in mouse models of mania (with hyperlocomotion caused by methylphenidate (5 mg/kg) or sleep deprivation for 24 h). After pre-treatment with (*R*)-(−)-carvone (50–100 mg/kg), (*S*)-(+)-carvone (50–100 mg/kg), and lithium (100 mg/kg positive control), the results showed that these doses do not modify spontaneous locomotor activity in the experiments induced by methylphenidate, whereas (*S*)-(+)-carvone caused a decrease in spontaneous locomotor activity in the experiment of sleep deprivation, which explains the induction of a sedative effect. Likewise, a 21-day chronic treatment with (*R*)-(−)-carvone (100 mg/kg), (*S*)-(+)-carvone (100 mg/kg), and lithium also allowed to block the hyperactivity induced by methylphenidate.

## 4. Conclusion and Perspectives

Here we have reported the benefits and pharmacological properties of carvone. This compound exhibited remarkable biological effects in vitro and in vivo and therefore may be a key candidate in drug development. Indeed, its anticancer and anti-inflammatory activities are promising with different mechanisms of action which allow us to consider it as a potential agent for the development of new anti-inflammatory and anticancer drugs. However, the pharmacodynamic actions were not well understood and, therefore, further investigations should be carried out to elucidate its mechanisms. Moreover, the study of combination between carvone used drugs in chemotherapy as well as its capacity to induce the sensitivity towards chemotherapy should be investigated. The antimicrobial action of carvone is also expected to be well determined in subsequent studies, and therefore further works should be investigated to determine its mechanism at subcellular, cellular, and molecular levels. Future clinical applications of carvone as an anticancer, anti-inflammatory, and antimicrobial drug could be developed in further investigations. However, these studies should take into account the validation of several steps. Indeed, the pharmacokinetic parameters should also be examined to determine the absorption, bioavailability, metabolism, and elimination of carvone. Moreover, toxicological investigations should be performed to validate its safety for other pharmaceutical applications.

## Figures and Tables

**Table 1:** Neurological activities of carvone.

Molecules	Origins	Models Used	Experimental Approaches	Key Results	References
(*S*)-(+)-Carvone and (*R*)-(−)-carvone	Purchased	Male Swiss mice	Pentobarbital-induced sleeping time Locomotor activity assessed in an activity cage PTZ-induced convulsions Pentobarbital-induced hypnosis PTZ-induced seizure PIC-induced seizure	LD_50_ = 484.2 mg/kg for (*S*)-(+)-carvone LD_50_ = 426.6 mg/kg for (*R*)-(−)-carvone Both enantiomers induced depressive effects Both enantiomers significantly reduced ambulation At 100 mg/kg, (*R*)-(−)-carvone was more effective than (*S*)-(+)-carvone in increasing pentobarbital sleeping duration At 200 mg/kg, (*S*)-(+)-carvone improved the latency of convulsions produced by PTZ and PIC (*S*)-(+)-carvone and (*R*)-(−)-carvone have depressant effects in the CNS (*S*)-(+)-carvone has anticonvulsant-like activity	[9]
(+)-carvone, (−)-carvone	Not reported	The sciatic nerve of the frog (*Rana ridibunda*) from both sex	Three-chambered recording bath for the assessment of local anesthetic activity	Both carvone enantiomers elicited comparable responses The action potential of the evoked compound was abolished in 6 to 7 min and had an immediate recovery of 83% to 87% Both carvones acted in the same way as lidocaine (10 mM) No recovery of the action potential of the elicited compound, when nerves have been exposed to carvones for more than 6–7 min The unusual neurotoxic effect of C+ and C− may be a disadvantage for their use in clinical practice	[10]
(+)-carvone, (−)-carvone	Purchased	Adult male Wistar rats	Sucrose-gap apparatus (ex vivo assay) for CAP-inhibitory effect	C- was less potent (IC_50_ = 10.7 ± 0.07 mM) in reducing nerve excitability than C+ (IC_50_ = 8.7 ± 0.1 mM) Both enantiomers acted in a similar manner The structure–function relationship of the enantiomers was linked to the CAP inhibitory action	[11]
(*R*)-(−)- carvone and (*S*)-(+)-carvone	Purchased	Cultures of cortical neurons prepared from the cerebral cortices of fetal rats	[^3^H] Flunitrazepam Binding Cell viability assay	Both isomers blocked GABA-induced activation of [^3^H] Flunitrazepam binding The doses required to produce negative receptor modulation were not lethal The insecticidal effect of carvones can be explained by their interaction with the GABAA receptor at its non-competitive blocker region	[12]

**Table 2:** Antidiabetic activity of carvone.

Molecules	Origins	Models Used	Experimental Approaches	Key Results	References
S-carvone	Purchased	C57BL/6 mice (male, ten weeks old)	GTT and ITT Histological examination Determination of hepatic triglyceride and serum lipid levels Determination of insulin resistance Gene expression analysis	Prevented weight gain, fat buildup in the liver, and insulin resistance Increased expression of macrophage marker genes in white adipose tissue, including *F4/80*, *Cd11b*, *Cd11c*, *Cd206*, and *Tnf-α* Decreased expression of genes involved for lipid production and transport in the liver (*Ppar2*, *Scd1*, *Cd36*) Inhibited high-fat diet-induced obesity and metabolic problems	[13]
Carvone	Purchased	Male Wistar rats weighing approximately 180–200 g	STZ-induced diabetes Estimation of blood glucose and plasma insulin levels Extraction and determination of glycoproteins	Improved glycemic status in a dose-dependent manner, in diabetic rats (30 mg/kg b.w.) Increased plasma insulin levels Reduced plasma glucose levels Restored the altered plasma and tissue glycoprotein levels Restored the abnormal levels of plasma and tissue glycoprotein components	[14]
Carvone	Purchased	Male Wistar rats (160–190 g)	STZ-induced diabetic rats Biochemical analysis Histopathological study of liver and pancreas Immunohistochemical examination of the pancreas	Decreased plasma glucose and HbA1c levels (50 mg/kg b.w.) Improved Hb and insulin levels Restored the reversed activity of carbohydrate metabolic enzymes, enzymic antioxidants, and hepatic marker enzymes Decreased STZ-induced damage to hepatic and pancreatic cells Controlled glucose metabolism by enhancing important enzymes in the hepatic tissues of diabetic rats	[15]

**Table 3:** Antifungal activity of Carvone.

Molecules	Origins	Strains Used	Experimental Approaches	Key Results	References
R-(−)-carvone	Purchased	Poly (lactic acid) (PLA) films for food packaging applications	Inclusion of R-(−)-carvone in the polymer matrix Preparation and determination of film thickness Determination of remaining content Determination of thermal, mechanical and barrier properties	Lower Tg and Tm Higher gas permeability Lower tensile strength Higher elongation at break of antifungal PLA films Homogeneous and transparent antifungal films	[16]
Carvone	Purchased	*Candida rugosa*, *Candida lusitaniae*, *Candida glabrata*, *Candida utilis*, *Candida krusei*, *Candida guilliermondii*, *Candida tropicalis*, *Candida albicans*, *Candida parapsilosis*, and *Candida dubliniensis*	Planktonic anti-candida assay Evaluation of the inhibitory power of germ tube formation Evaluation of the anti-biofilm effect	MIC = 0.5 mg/mL The concentration of 0.5 mg/mL inhibited at least 50% of the biofilm Inhibited the polymorphism up to 86% Changes in yeast cell envelope and cell viability were greater than 50% Induced important antifungal activities	[17]
Carvone chemotype	Naturel	*Candida parapsilosis*, *Candida krusei*, *Aspergillus flavus*, and *Aspergillus fumigatus* Broth macro-dilution method AFST-EUCAST method CLSI M38-A method MIC determination	Determination of GM-MIC	GM-MIC > 500 μg/mL against the different strains studied No activity against selected clinical strains	[18]
Carvone	Purchased	*Fusarium subglutinans*, *Fusarium cerealis*, *Fusarium verticillioides*, *Fusarium proliferatum*, *Fusarium oxysporum*, *Fusarium sporotrichioides*, *Aspergillus tubingensis*, *Aspergillus carbonarius*, *Alternaria alternata*, and *Penicillium* sp.	In vitro antifungal activity Evaluation of deoxynivalenol production Evaluation of inhibitory effects on plant seed germination	Induced toxic effects on the growth of the mycelium of all fungal species	[19]
Carvone	Naturel (*Mentha spicata*)	*Cryptococcus neoformans*, dermatophytes (*Trichophyton* spp., *Epidermophyton floccosum*, and *Microsporum* spp.), and *Aspergillus* strains	In vitro antifungal activity Evaluation of the inhibitory activity of germ tube formation	*Mentha spicata* EO was effective against *Cryptococcus neoformans*, as well as the dermatophytes *Trichophyton rubrum* and *Trichophyton verrucosum* (0.32 μL/mL) Inhibited the germ tube development of *Candida albicans*, at concentrations below the MIC (0.16 μL/mL)	[21]
(+)-carvone (C+) (−)-carvone (C−) α,β-epoxycarvone (EP) (+)-hydroxy-dihydrocarvone (HC+) (−)-hydroxy-dihydrocarvone (HC−)	Purchased	*Candida parapsilosis*, *Candida tropicalis*, *Candida krusei*, and *Candida albicans*	Determination of MIC by microplate dilution method and MFC	Low antifungal activity against *Candida tropicalis* and *Candida parapsilosis* EP and C+ showed moderate activity against *Candida krusei* similar to C+ and C− against *Candida albicans* All the molecules tested showed fungistatic and fungicidal activity against *Candida* yeasts, and the most significant result was recorded with C+, C−, and EP	[20]

**Table 4:** Antibacterial activity of carvone.

Molecules	Origins	Model Used	Experimental Approaches	Key Results	References
(*S*)-(−)-carvone (*R*)-(+)-carvone	Naturel (*Mentha spicata* and *Anethum sowa* Roxb.)	*Bacillus subtilis*, *Enterobacter aerogenes*, *Enterococcus Faecalis*, *Klebsiella pneumoniae*, *Pseudomonas aeruginosa*, *Staphylococcus aureus*, *Streptococcus mutans*, *Yersinia enterocolitica*, *Salmonella typhi*, *Escherichia coli*, *Staphylococcus epidermidis*, and *Mycobacterium smegmatis*	Disk diffusion assay Broth dilution assay	The activity of carvone was comparable with the bioactivity of their original oils Active against a broad spectrum of human pathogenic bacteria (R)-(+)-limonene showed comparable bioactivity profile over the (S)-(−)-isomer	[22]
Carvone	Purchased	*Staphylococcus aureus*	Single-step plasma polymerization Plasma polymerization of carvone Surface characterization Antibacterial activity Live-dead fluorescence assay Crystal violet assay Morphology of bacteria by field emission scanning electron microscope (FE-SEM)	Polymerization provided a hydrophobic antibacterial coating (ppCar) with an average roughness < 1nm ppCar had a static water contact angle of 78° Reduced effectively *Escherichia coli* (86%) and *Staphylococcus aureus* (84%) Broken bacterial membrane	[23]
(−)-Carvone (+)-Carvone	Purchased	*Absidia glauca*, *Staphylococcus aureus*, *Escherichia coli, Pseudomonas aeruginosa, Enterobacter aerogenes, Proteus vulgaris,* and *Salmonella typhimurium*	Biotransformation Semi-preparative scale biotransformation and isolation GC-MS Antimicrobial assay	Biotransformation of carvone into diol 10-hydroxy-(+)-neodihydrocarveol by *Absidia glauca* Both molecules showed antimicrobial activity against all strains tested	[24]
Semicarbazone and thiosemicarbazone of R-(−) carvone	Synthetized	*Escherichia coli*, *Staphylococcus aureus*, *Pseudomonas aeruginosa*, and *Enterococcus faecalis*	Determination of MIC	Inhibitory activity on *Pseudomonas aeruginosa* for thiosemicarbazone (MIC = 78.1 μg/mL) and for semicarbazone (MIC = 312.5 μg/mL) Thiosemicarbazone was active on *Staphylococcus aureus* (MIC = 39 μg/mL) Thiosemicarbazone exerted interesting inhibitory activity on *Staphylococcus* *aureus* and *Pseudomonas* *aeruginosa*	[26]
Carvone	Purchased	*Staphylococcus aureus* and *Enterococcus coli*	Nanoparticles preparation Determination of drug loading and entrapment efficiency In vitro carvone release from nanoparticles Antibacterial properties of the carvone-loaded nanoparticles	Production of small nanoparticles (126 nm), with high drug loading (12.32%) and good inhibition of microbial growth Carvone-loaded nanoparticles inhibited *Staphylococcus aureus* (MIC = 182 mg/mL) and *Enterococcus coli* (MIC = 374 mg/mL)	[25]
(+)-carvone (−)-carvone (+)-hydroxy-dihydrocarvone (−)-hydroxyl-dihydrocarvone α,β-epoxycarvone	Synthesized/purchased	*Escherichia coli* and *Staphylococcus aureus*	Determination of MIC by microplate dilution method and MBC	C- and HC- showed low activity against *Escherichia coli* EP, C+, and HC+ did not inhibit the growth of the bacterial strains tested	[20]
R-carvone S-carvone	Purchased	Methicillin-resistant *Staphylococcus aureus* (MRSA)	Broth micro-dilution method Time-kill assay	MIC values for R- and S-carvone against six different strains of *Staphylococcus aureus* ranged between 500 and 1000 µg/mL R-carvone + gentamicin and S-carvone + gentamicin exhibited significant synergistic activity against MRSA The combined treatment improved the effectiveness of carvone	[27]
Carvone	Naturel (*Lippia alba*)	*Staphylococcus aureus* ATCC 6538	Determination of MIC and MBC by the microdilution method Anti-biofilm Activity	Elimination of biofilm cells was confirmed at concentrations between 0.5 and 2 mg/mL No elimination of biofilm cells was observed with the use of carvone	[28]

**Table 5:** Antiviral activity of carvone neuraminidase.

Molecules	Models Used	Experimental Approaches	Key Results	References
Two analogues of carvone	In silico study	Molecular docking Molecular dynamics simulation	All ligands showed strong binding affinity against active neuraminidase sites, ranging from −4.77 to −8.30 kcal/mol Carvone derivatives could serve as potent neuraminidase inhibitors against the influenza virus	[38]

## Data Availability

Not applicable.

## Abbreviations

Bcl-2	B-cell lymphoma 2
BITC	Benzyl isothiocyanate
BZD	Benzodiazepines
CAP	Compound action potential
CDK1	Cyclin-dependent kinase 1
CNS	Central nervous system
CV	Crystal violet
DPPH	2,2-dipenyl-1-picrylhydrazyl
EO	Essential oil
ESE	Evaporation solvent
FE-SEM	Field emission scanning electron microscope
GC-MS	Gas chromatography–mass spectrometry
GM-MIC	Geometric means–minimal inhibitory concentration
GTT	Glucose tolerance test
Hb	Hemoglobin
HbA1c	Glycosylated hemoglobin
IC50	Half-maximal inhibitory concentration
IFN-γ	Interferon gamma
IL	Interleukin
ITT	Insulin tolerance test
LC50	Lethal concentration 50%
LD50	Medium lethal dose
LPS	Lipopolysaccharide
MBC	Minimum bactericidal concentration
MFC	Minimum fungicidal concentration
MIC	Minimum inhibitory concentration
MRSA	Methicillin-resistant staphylococcus aureus
MS	Mass spectrometry
NMR	Nuclear magnetic resonance
PARP	Poly adenosine diphosphate-ribose polymerase
PGE2	Prostaglandin E2
PLA	Poly (lactic acid)
PLGA	Poly (lactic-co-glycolic acid)
ROS	Reactive oxygen species
SIRT1	Sirtuin-1
STZ	Streptozotocin
TBARS	Thiobarbituric acid reactive species
TLC	Thin layer chromatography
TNF-α	Tumor necrosis factor-α
